# The onset of coarctation of the aorta before birth: Mechanistic insights from fetal arch anatomy and haemodynamics

**DOI:** 10.1016/j.compbiomed.2024.109077

**Published:** 2024-11

**Authors:** Uxio Hermida, Milou P.M. van Poppel, Malak Sabry, Hamed Keramati, Johannes K. Steinweg, John M. Simpson, Trisha V. Vigneswaran, Reza Razavi, Kuberan Pushparajah, David F.A. Lloyd, Pablo Lamata, Adelaide De Vecchi

**Affiliations:** a Department of Biomedical Engineering, School of Biomedical Engineering and Imaging Sciences, King's College London, St Thomas' Hospital, London, SE1 7EH, UK; b Department of Cardiovascular Imaging, School of Biomedical Engineering and Imaging Sciences, King's College London, St Thomas' Hospital, London, SE1 7EH, UK; c Department of Perinatal Imaging, School of Biomedical Engineering and Imaging Sciences, King's College London, St Thomas' Hospital, London, SE1 7EH, UK; d Department of Congenital Heart Disease, Evelina London Children's Hospital, SE1 7EH, UK

**Keywords:** Computational fluid dynamics, Digital twin, Fetal magnetic resonance imaging, Statistical shape model, Wall shear stress

## Abstract

Accurate prenatal diagnosis of coarctation of the aorta (CoA) is challenging due to high false positive rate burden and poorly understood aetiology. Despite associations with abnormal blood flow dynamics, fetal arch anatomy changes and alterations in tissue properties, its underlying mechanisms remain a longstanding subject of debate hindering diagnosis in utero. This study leverages computational fluid dynamics (CFD) simulations and statistical shape modelling to investigate the interplay between fetal arch anatomy and blood flow alterations in CoA. Using cardiac magnetic resonance imaging data from 188 fetuses, including normal controls and suspected CoA cases, a statistical shape model of the fetal arch anatomy was built. From this analysis, digital twin models of false and true positive CoA cases were generated. These models were then used to perform CFD simulations of the three-dimensional fetal arch haemodynamics, considering physiological variations in arch shape and blood flow conditions across the disease spectrum. This analysis revealed that independent changes in the shape of.

the arch and the balance of left-to-right ventricular output led to qualitatively similar haemodynamic alterations. Transitioning from a false to a true positive phenotype increased retrograde flow through the aortic isthmus. This resulted in the appearance of an area of low wall shear stress surrounded by high wall shear stress values at the flow split apex on the aortic posterior wall opposite the ductal insertion point.

Our results suggest a distinctive haemodynamic signature in CoA characterised by the appearance of retrograde flow through the aortic isthmus and altered wall shear stress at its posterior side. The consistent link between alterations in shape and blood flow in CoA suggests the need for comprehensive anatomical and functional diagnostic approaches in CoA. This study presents an application of the digital twin approach to support the understanding of CoA mechanisms in utero and its potential for improved diagnosis before birth.

## Highlights

• Independent shape and flow changes lead to a similar haemodynamic phenotype in CoA.
• Retrograde aortic isthmus flow and a TAWSS split pattern as key features of interest.
• The link between shape and flow support CoA aetiology theories.
• Digital twin approaches have potential to improve understanding of CoA before birth.
• Digital twin approaches have potential for discovery of novel biomarkers.

## Graphical abstract

**Figure:**
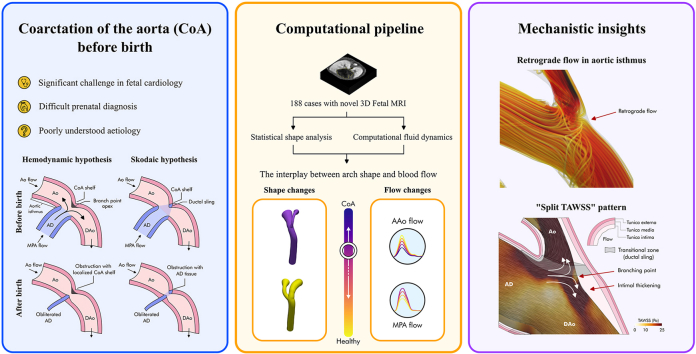

## Abbreviations:

AAo: Ascending aorta AD: Arterial duct
CoA: Coarctation of the aorta
CFD: Computational fluid dynamics
CMR: Cardiac magnetic resonance imaging DAo: Descending aorta
FP: False positive
LDA: Fisher Linear Discriminant Analysis LV: Left ventricular
MPA: Main pulmonary artery
PCA: Principal Component Analysis
PC-MRI: Phase-contrast magnetic resonance imaging RV: Right ventricular
SSM: Statistical shape modelling TAWSS: Time-averaged wall shear stress WSS: Wall shear stress
2D: Two-dimensional 3D: Three-dimensional
3WK: Three-element Windkessel model

## 1 Introduction

Neonatal coarctation of the aorta (CoA) is a common form of congenital heart disease characterised by the constriction of the aortic isthmus after the arterial duct closes following birth. The diagnosis is suspected with fetal ultrasonography when disproportion in the size of the ventricles, great arteries, arterial duct and aortic arch is present. With this approach, there are high false positive (FP) rates between 50 and 90 % [1,2]. Measurements of the arterial valves, arterial duct and aortic arch may reduce these FP rates [3], but accurate prenatal diagnosis remains challenging. Additionally, the underlying aetiological factors and pathophysiological mechanisms governing CoA are yet to be fully elucidated, contributing to the complexity of diagnosis.

**Fig. 1:**
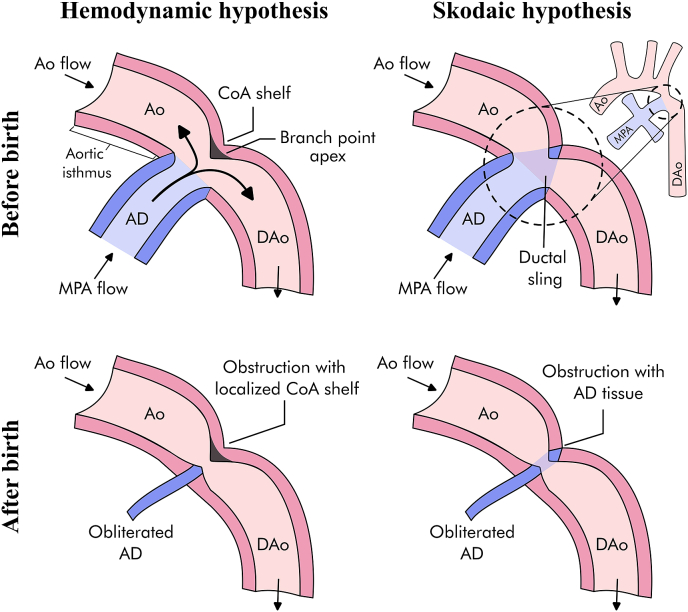
Main mechanistic theories of coarctation of the aorta (CoA) onset before birth and after birth. The left panel illustrates the haemodynamic hypothesis, proposing that CoA results from abnormal haemodynamic changes during fetal development. Specifically, the division of ductal flow at the arterial junction (branch point apex) is believed to lead to the formation of the CoA shelf, causing postnatal arterial constriction. The right panel displays the Skodaic Theory, which suggests that CoA occurs due to the abnormal extension of arterial duct cells into the aorta (ductal sling), resulting in postnatal narrowing. AD - Arterial duct; Ao - Aorta; CoA - Coarctation of the aorta; DAo - Descending aorta; MPA - Main pulmonary artery.

The aetiology of CoA has been a longstanding subject of debate, with two main mechanistic theories: the haemodynamic theory [4,5], and the Skodaic theory [6] (see Fig. 1). The haemodynamic hypothesis, or branching theory, suggests that CoA arises from anomalous haemodynamic changes during fetal development, which result in the aortic isthmus becoming a functional branch of the arterial duct. Hutchins [4] observed histological similarities between the posterior aortic isthmus shelf in CoA, which is the ridge of infolded tissue associated with its obstruction after the postnatal closure of the arterial duct, and other major vascular bifurcations. He also suggested that intracardiac defects such as bicuspid aortic valve or aortic stenosis may cause right ventricular dominance (and thus alter aortic and ductal flow), and this, in turn, might contribute to the arterial remodelling in CoA. Similarly, Rudolph et al. [5] emphasised the relevance of anatomical features, such as the angle at which the arterial duct joins the aorta, and the presence of retrograde flow in the aortic isthmus for vascular remodelling in CoA.

In contrast, the Skodaic theory [6] postulates that CoA results from the abnormal extension of arterial duct cells into the aorta, which, compared to aortic cells, show structural properties similar to those of muscular arteries, such as disassembly and fragmentation of the internal elastic lamina and sparse elastic fibers in the middle layer. After birth, the rapid decline in circulating prostaglandin E2 (PGE2) that triggers the obliteration of the arterial duct could lead to the observed postnatal constriction in CoA as PGE2 inhibits elastogenesis via EP4 signalling in ductal cells [7]. This theory has been backed up by several histological studies reporting increased circular or partial distribution of arterial duct media in aortic tissues, often referred to as ductal sling. Histological findings have also shown that the migration of smooth muscle cells from the ductal media to the intimal layer contributes to the characteristic intimal thickening observed in CoA [[8], [9], [10], [11]].

Recent research has highlighted the relevance of the 3D fetal arch anatomy for the prediction of CoA before birth, as well as its potential links with the CoA pathophysiology [12,13]. Additionally, a recent study [14] showed initial evidence of the 3D fetal arch haemodynamic differences between false and true positive CoA cases using

Computational fluid dynamics simulations. It is known that changes in blood flow, pressure and wall shear stress (WSS) can lead to adverse vessel remodelling, impacting vessel calibre, shape and stiffness. Importantly, these factors can also affect endothelial cell behaviour (e.g., cell migration, survival, proliferation or alignment), altering the arterial tissue structure. These changes can exacerbate the adverse vessel remodelling, which in turn can lead to altered haemodynamic conditions, perpetuating this vicious cycle. However, the inability of traditional diagnostic methods, such as imaging, to identify the interplay between shape and flow changes means the signature of CoA remains unknown and thus prenatal diagnosis particularly challenging.

A digital twin is an in-silico replica of a real-world system (the heart in this case) that is driven by data and has the ability to generate specific predictions of biomarkers or outcomes. In this context, a Digital Twin based on fetal CMR imaging that integrates statistical shape modelling and computational fluid dynamics (CFD) simulations to study the effect of independent fetal arch shape and flow changes in CoA could shed light on their mechanistic link and their relevance in the CoA pathophysiology. However, the application of CFD to investigate 3D fetal arch haemodynamics has been limited to date due to data scarcity [[14], [15], [16]]. Although some studies have explored specific aspects of fetal arch haemodynamics using idealised conditions, none have comprehensively examined the interplay between blood flow dynamics and anatomical remodelling in CoA before birth, particularly considering physiological flow and shape alterations.

This study aims to address this knowledge gap by building computer replicas of the fetal aorto-ductal system using a unique dataset of 3D fetal cardiac MRI data to gain novel insights into the key mechanisms driving CoA in utero.

## 2 Materials and methods

**Fig. 2:**
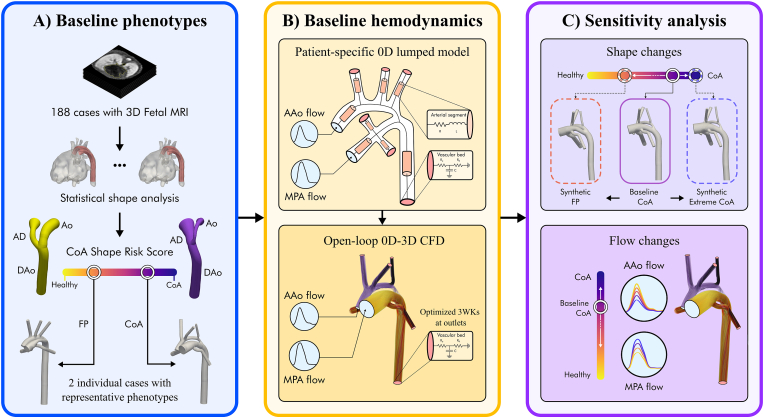
Computational modelling pipeline. First, a statistical shape analysis of a set of fetal arch anatomies is used to select two individual representative cases: one false positive (FP) and one confirmed coarctation of the aorta (CoA)- **Panel A** After the cases with representative shape phenotypes are selected, the baseline haemodynamics are derived based on flow from 2D phase-contrast magnetic resonance imaging. A 0D lumped parameter model is used to tailor the flow boundary conditions in each vessel to the magnetic resonance imaging data. These 0D boundary conditions are then coupled to the 3D models of the two selected cases for computational fluid dynamics simulations- **Panel B.** After the completion of the baseline simulations, physiological alterations of blood flow and shape are applied to each model to perform a sensitivity analysis of the interplay between haemodynamics and anatomical changes - **Panel C.** Ao - Aorta; AAo - Ascending aorta; AD - Arterial duct; CoA - Coarctation of the aorta; DAo - Descending aorta; 3WK - Three-element Windkessel model; LDA - Fisher Linear Discriminant Analysis; MPA - Main pulmonary artery; PCA - Principal Component Analysis.

We study the interplay between fetal arch anatomy and haemodynamics by performing in-silico CFD simulations where either the flow or shape conditions are perturbed. Perturbations are introduced over baseline flow and shape conditions adjusted to patient characteristics. An overview of our modelling pipeline is illustrated in Fig. 2. First, two individual representative cases of confirmed and false positive CoA (one each) were selected based on a statistical shape analysis of fetal arch anatomies that quantifies the anatomical features associated with CoA (Fig. 2A). Subsequently, the baseline 3D haemodynamics in the fetal arch were derived for each representative case from MRI data (Fig. 2B). After completion of these baseline simulations, physiological perturbations of the blood flow and shape were introduced to perform a sensitivity analysis of the interplay between haemodynamics and anatomical changes (Fig. 2C).

### 2.1 Patient selection and study design

A total of 188 fetal cardiovascular magnetic resonance (CMR) data were acquired at Evelina London Children's Hospital between 2015 and 2023. The study population comprised 62 healthy control cases (median gestational age (GA): 30.5 weeks; interquartile range: 29.7–31.9) and 126 cases with suspected CoA. Among these, 48 cases were confirmed as true CoA, or true positives (TP) (median GA: 32.0 weeks; interquartile range: 30.9–33.4), and 78 were false positives (FP) (median GA: 31.9 weeks; interquartile range: 30.9–32.9). Exclusion criteria included maternal weight exceeding 125 kg, claustrophobia, inability to give informed consent, or being under the age of 18 years. Cases with external compression on left heart structures or anomalies in ventriculoarterial connections were not included in the analysis. Informed consent for fetal CMR research participation was obtained for all patients as part of the Intelligent fetal Imaging and Diagnosis (iFIND) Project (Research Ethics Committee (REC) no:14/LO/1806) or Quantification of fetal growth and development using MRI (REC no: 07/H0707/105). This study does not contain any studies with animals performed by any of the authors.

The CMR data was acquired using a 1.5 T Ingenia MR system (Philips, Best, the Netherlands). CMR examinations consisted of multiple T2-weighted 3D black-blood standard single-shot fast spin echo sequences covering the fetal thorax, with repetition time (TR) = 20,000 ms, echo time (TE) = 80 ms, flip angle = 90°, voxel size = 1.25 × 1.25 mm, slice thickness = 2.5 mm, sensitivity encoding (SENSE) factor = 2, partial Fourier factor = 0.547, and slice duration = 546 ms. No external gating, fetal or maternal sedation, or intravenous contrast were used. 2D stacks were transformed into high-resolution 3D volumes (range 0.55–0.75 mm isotropic) using Slice-to-Volume Registration (SVR) [17,18] and Deformable SVR [19,20].

Segmentations of the fetal heart were performed using ITK-SNAP (version 3.6.0) with manual refinements by 3 clinicians with fetal CMR experience independently (M.P.M.v.P, D.F.A.L, J.K.S). All reconstructions and segmentations were inspected before inclusion. Data also included 2D Phase-Contrast MRI retrospectively gated using metric optimised gating, from which flow reconstruction was performed [[21], [22], [23]]. Data were acquired in six fetal vessels: ascending aorta (AAo), descending aorta (DAo), superior vena cava, main pulmonary artery (MPA), arterial duct and umbilical vein. Data processing was done with CVI42 (Circle Cardiovascular Imaging, Calgary, Version 5.6.4) using semi-automated contouring techniques integrated into the software following an internally validated protocol. No background correction was applied. The quality score previously published in Ref. [23] was used to assess the quality of the fetal flow sequences for CFD simulations before inclusion.

### 2.2 Baseline phenotypes: statistical shape model

The selection of two individual cases with representative shape phenotypes was based on a statistical shape model (SSM) of the fetal arch, which captures common shape changes within a population and facilitates the exploration of characteristic disease-associated phenotypes.

The construction of this SSM followed the methodology outlined in Ref. [13]. In brief, we extracted centreline points and radii from the ascending aorta (AAo), descending aorta (DAo) and arterial duct to encode the 3D fetal arch shape. After alignment into a common reference space, Principal Component Analysis (PCA) was used to build the SSM, resulting in a set of linear axes that capture the most common shape changes in the cohort (i.e., PCA modes). PCA modes are ranked based on the amount of variance explained so that the first modes represent the most robust shape features. The first 10 PCA modes were then used with a Fisher Linear Discriminant Analysis (LDA) to find the anatomical axis that best distinguishes between FP and true CoA cases - essentially capturing the disease spectrum from healthy to CoA [24].

As a result, each case was characterised by a CoA shape risk score (Z-score) along the LDA axis. The shape score was used to select the two individual cases with representative shape phenotypes. We selected a confirmed true positive CoA case with a shape score near the average of the confirmed CoA population (gestational age 30.3 weeks) and a FP case (gestational age 31.3 weeks) with a shape close to the healthy side of the disease spectrum (CoA shape score near the average of the healthy controls).

For the CFD simulations, 3D anatomies were reconstructed using a linear combination of these 10 PCA modes with the addition of the right and left pulmonary arteries, as well as the arterial segments extending to the upper body and brain. This ensures that spurious shape features are not included in the models. For more details about the reconstruction of 3D anatomies from PCA modes, see Supplementary Materials.

### 2.3 Baseline haemodynamics: open-loop 0D-3D CFD

Open-loop 0D-3D models were built following the approach detailed in Ref. [14]. First, three-element Windkessel models (3WK) were calibrated based on 2D PC-MRI flow data for each outlet using a case-specific 0D lumped model of the

fetal circulation. Each 3WK model consisted of a proximal resistance in series with a parallel combination of a distal resistance and a capacitor, to capture the Windkessel effect typical of arterial segments subjected to physiological pulsatile flow. The flow rate waveforms derived from 2D PC-MRI were used as inflow boundary conditions in the ascending aorta and main pulmonary artery. Then, the cost function for 3WK optimisation consisted of a weighted combination of relative errors, including flow rate and time-to-peak velocity at the descending aorta, the distribution of combined cardiac output (CCO) to each outlet, and the theoretical systolic and diastolic blood pressures from Ref. [25]. For more details about the 0D lumped model of the fetal circulation and its implementation, see Supplementary Materials.

All open-loop 0D-3D simulations were performed using the finite volume solver Ansys Fluent 2021 R2 (Ansys Inc. USA) until periodic conditions were achieved. The velocity waveforms derived from 2D PC-MRI were imposed at the model inlets with a plug velocity profile (AAo and MPA, Fig. 2B). The calibrated 3WK models were applied to each outlet. Blood flow was assumed to be laminar and modelled as an incompressible Newtonian fluid with a density of 1060 kg/m^3^ and viscosity of 3.6 mPa s [26]. All simulations were performed using an unstructured mesh with 9 · 10^5^ to 1 · 10^6^ tetrahedral elements and average element volume of 2.1 · 10^−12^ to 2.4 · 10^−12^ *m*^3^. The boundary layer was generated using 5 layers, with a growth rate of 1.2 and a transition ratio of 0.272 (average boundary layer element volume 1.8 · 10^−12^ *m*^3^). All simulations were performed with a time-step of 1 · 10^−4^ *s*. For more information about the mesh and time-independence studies, see Supplementary Materials.

### 2.4 Sensitivity analysis

The sensitivity analysis was performed in two separate experiments as follows.

#### 2.4.1 Shape changes

The LDA axis Φ_*LDA*_ defines a direction of anatomical remodelling from a healthy to a diseased CoA state centred on the population's average. This direction can be used to manipulate individual anatomies *x*_*i*_ and generate new synthetic anatomies *x*_*i*_’ aligned with the disease axis using

**Fig. 3:**
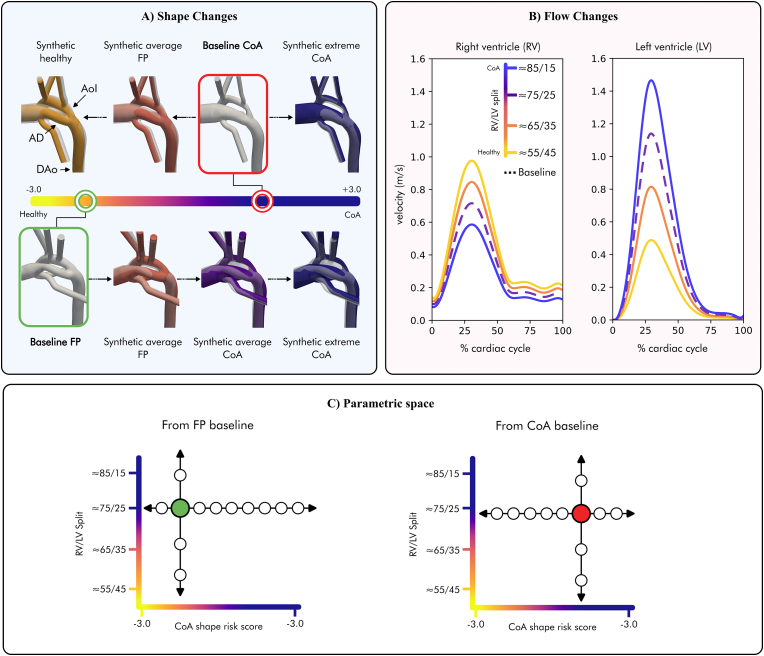
Experimental setup for the sensitivity analysis of the interplay between shape changes and blood flow changes. **Panel A** shows the generation of synthetic anatomies from two cases with representative baseline phenotypes (boxed) spanning the disease spectrum. **Panel B** shows the different inlet boundary conditions used for the computational fluid dynamics simulations. AD - Arterial duct; AoI - Aortic isthmus; CoA - Coarctation of the aorta; FP - False positive; LV - Left ventricle; RV - Right ventricle.

(1) *x*_*i*_′ = *x*_*i*_ + *α* · Φ_*LDA*_

where *α* is the control parameter governing both the extent and the direction of anatomical changes. The values along the LDA axis span from −3 standard deviations (SD) to +3SD, denoting the Z-score range. For a comprehensive assessment of the effect of shape changes on the baseline haemodynamics in the two selected cases with representative phenotypes, synthetic anatomies were sampled in increments of 0.5SD to build an in-silico cohort covering the spectrum of disease. For the FP case, since its initial baseline shape score was closer to the healthy population average, only positive *α* values were chosen, from 0 to 4.0 in increments of 0.5SD. For the CoA case, *α* values ranged from −3.0 to 1.0SD, to allow exploration of both healthy, FP and extreme CoA synthetic anatomies. Fig. 3A shows graphically the generation of synthetic anatomies from the two baseline phenotypes spanning the disease spectrum.

#### 2.4.2 Flow changes

We explored the influence of haemodynamic changes in CoA focusing on the case-specific balance between right ventricular (RV) and left ventricular (LV) inlet boundary conditions. In CoA, fetuses exhibit significant left-to-right volume redistribution and associated biventricular remodelling [27,28]. While RV dominance during fetal life is normal, ventricular disproportion is exacerbated in cases with suspected CoA, with true positive CoA cases displaying an even more pronounced disparity than false positive cases [12]. The volume redistribution towards the RV is thought to be a compensation mechanism that allows the heart to provide most of the lower body flow through the RV, thus protecting the LV from having to eject through the narrow aorta, which would result in an increase in the pressure load. Furthermore, alterations in the aortic isthmus size have been linked to changes in cardiac output distribution [12].

In the selected CoA case, the baseline distribution of cardiac output was 72 % through the RV, while 28 % from the LV. Using this as a starting baseline, a set of simulations with different left-to-right ventricular output splits were explored, covering the whole haemodynamic disease spectrum (CoA to healthy): 82/12 %, 72/28 %, 62/38 %, 52/48 %. In the FP case, the baseline flow split was 75/25 % and the following scenarios were simulated: 85/15 %, 75/25 %, 65/35 %, 55/45 %. All simulations were performed using the baseline 3D shapes. Fig. 3B shows an example of the different inlet boundary conditions used for the CFD simulations.

A total of 24 0D-3D open-loop simulations were performed for the sensitivity analysis to explore the effect of shape and flow changes on the baseline haemodynamic conditions, comprising 2 baseline simulations with baseline shape and flow, 6 simulations with baseline shape and changing flow conditions, and 16 simulations with changing shape and baseline flow conditions. The distribution of time-averaged wall shear stress (TAWSS) along the fetal arch and the streamlines flow pattern were assessed.

## 3 Results

### 3.1 Statistical shape model

The first 10 PCA modes captured 87 % of the shape variability in the population. Their linear combination and resulting CoA shape risk score was able to classify each class with excellent area under the receiver operating characteristic curve (AUC): 0.92 between false positives and confirmed CoA cases; 0.86 between false positives and controls, and 1.0 between controls and confirmed CoA cases.

### 3.2 Baseline characteristics of the FP and CoA cases

**Fig. 4:**
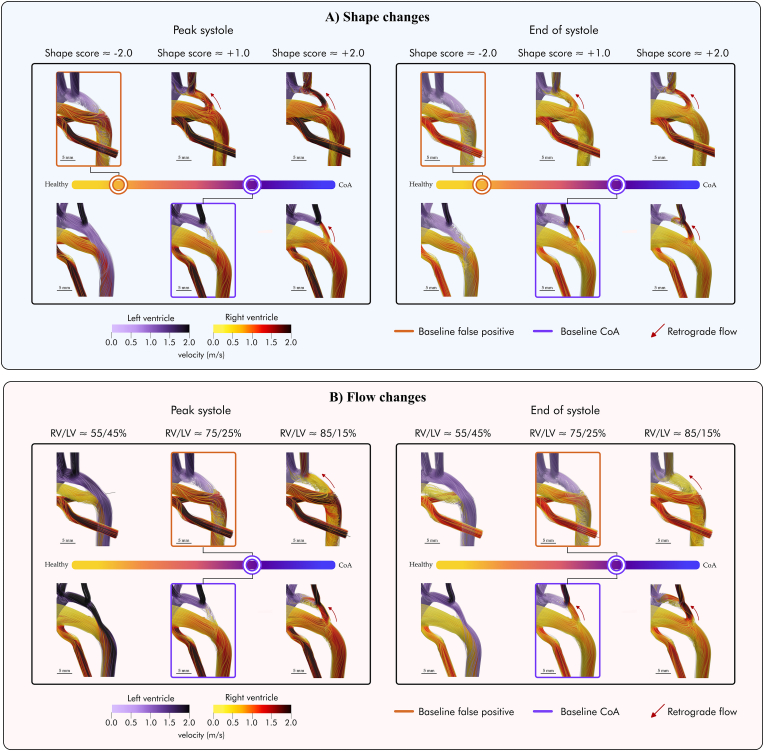
**Panel A** shows the impact of shape changes from the baseline simulations for the n = 2 studied cases with representative phenotypes, false positive (FP - orange box) and true positive coarctation of the aorta (CoA - purple box), spanning the disease spectrum captured by the shape score from healthy to diseased (i.e., from −2 standard deviations (SD) to +2SD). From left to right, the anatomy changes from a more healthy shape (yellow) to a more CoA one (purple). **Panel B** shows how variations in inlet boundary conditions influence the baseline haemodynamics (boxed) for the two studied shape phenotypes. Red arrows show the appearance of retrograde flow in the aortic isthmus. Two different colour maps are used to highlight the origin of the blood flow - left ventricle or right ventricle. AD - Arterial duct; AoI - Aortic isthmus; CoA - Coarctation of the aorta; DAo - Descending aorta; LV - Left ventricle; RV - Right ventricle.

In the FP case, the flow from the arterial duct met the aortic isthmus flow laterally forming a helical pattern from peak systole to end-systole (see dashed box in Fig. 4A). Conversely, in the CoA case, the arterial duct flow entered

the descending aorta without a helical pattern (see dashed box in Fig. 4A).

In the FP case, there was no retrograde flow through the isthmus. However, the CoA case displayed a physiologi-cal retrograde flow during the systolic deceleration phase (see dashed line in Fig. 4B). Minimal retrograde flow in the aortic isthmus was observed during peak systole in this CoA case.

Differences in time-averaged wall shear stress (TAWSS) were observed between the two phenotypes. The FP case showed increased TAWSS in the arterial duct compared to the confirmed CoA case (maximum TAWSS 62.6 Pa vs 30.2 Pa). In the aortic isthmus, the FP showed reduced TAWSS compared to the confirmed CoA case (mean TAWSS 3.3 ± 3.8 vs 9.2 ± 5.4 Pa; maximum TAWSS 23.1 vs 40.0 Pa).

### 3.3 Shape changes

In the first experiment, independently changing the vascular anatomy induced haemodynamic changes consistently across the two cases with representative shape phenotypes. Fig. 4A illustrates the impact of anatomical variations on baseline haemodynamics. Table 1 shows the results at the aortic isthmus when moving along the shape axis.

**Table 1:** Comparison of the time-averaged wall shear stress (TAWSS) and flow rate *Q* at the aortic isthmus when moving along the shape axis.

	Shape changes - Shape score				
	**−2.0**	**−1.0**	**0.0**	**1.0**	**2.0**
**TAWSS**	**5.0** ± **2.9**/	**5.6** ± **3.3**/	**9.2** ± **5.4**/	**9.2** ± **5.4**^b^/	**21.0** ± **10.1**/
	3.3 ± 3.8^a^	5.2 ± 3.5	9.6 ± 6.3	19.6 ± 11.0	27.4 ± 15.8
TAWSS_*max*_	**16.8**/23.1^a^	**20.3**/20.6	**38.2**/27.7	**40.0**^b^/41.2	**48.4**/82.1
**Q**_*ist*_	**108.0**/67.5^a^	**73.1**/52.5	**49.1**/66.8	**42.7**^b^/85.6	**54.0**/85.8
**Q**_*istretrograde*_	**−5.2**/-1.7^a^	**−9.2**/-21.2	**−18.2**/−47.8	**−33.5**^b^/-72.5	**−51.8**/-79.5

The bold values correspond to the simulations starting from the true positive baseline. TAWSS shows the mean standard deviation of the aortic isthmus region. TAWSS_*max*_ the maximum TAWSS. Q_*ist*_ the total flow rate through the aortic isthmus. Q_*istretrograde*_ the retrograde flow rate. TAWSS values are shown in Pa; *Q* values in mL/min.

a Shows the false positive baseline results.

b The true positive baseline ones.

**Fig. 5:**
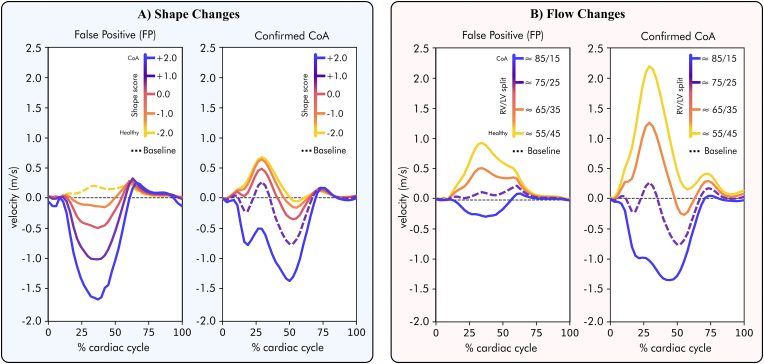
Variations in the aortic isthmus velocity profile depending on the shape (**Panel A**) and inlet flow changes (**Panel B**) covering the disease spectrum from healthy (yellow) to diseased (purple) conditions. The baseline conditions are shown with dashed lines. CoA - Coarctation of the aorta; LV - Left ventricle; RV - Right ventricle.

Fig. 5A illustrates the velocity waveforms at the aortic isthmus for each of the simulated anatomies, showing how the progressive changes in shape resulted in increased or decreased retrograde isthmus flow. Anatomical changes towards a more CoA-like shape resulted in gradually diminished antegrade flow through the aortic isthmus, eventually leading to retrograde flow through it towards the left subclavian carotid artery in CoA anatomies (indicated by red arrows in Fig. 4A.). They also shifted the peak of reverse flow earlier in the cardiac cycle, particularly in the confirmed CoA case. On the other hand, progressive changes towards a healthier anatomy resulted in the opposite, with increased antegrade flow through the aortic isthmus. Ultimately, this led to the absence of retrograde flow, both at peak and end-systole (Fig. 4, Fig. 5A).

**Fig. 6:**
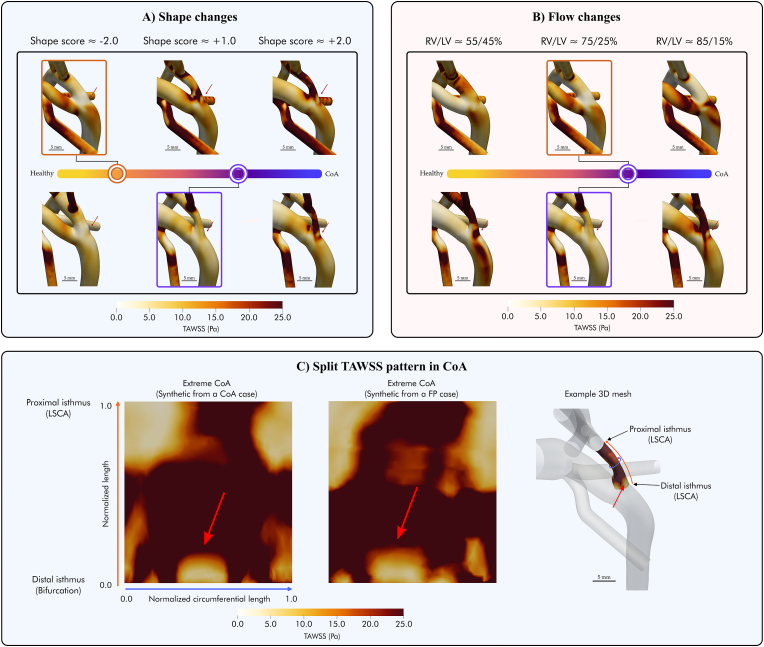
Variations in time-averaged wall shear stress (TAWSS) as a result of fetal arch shape (**Panel A**) and inlet blood flow changes (**Panel B**). Red arrows in the posterior wall of the aortic isthmus (AoI) show the progressive appearance of the “split TAWSS pattern” as the shape evolves to that of the CoA, i.e. a low TAWSS spot surrounded by high TAWSS. **Panel C** shows the “TAWSS split pattern” in: the extreme CoA shape from the false positive baseline shape - right case in the top row Panel A; the extreme CoA shape from the true positive CoA baseline anatomy - right case in the bottom row in Panel A. The 3D mesh shows the correspondence between the flattened maps on the left and the 3D meshes. The flattened maps are a normalized representation of the aortic isthmus. The purple line shows the radial length in both the flattened map and the 3D mesh, and the orange line the aortic isthmus length. AoI - Aortic isthmus; CoA - Coarctation of the aorta; DAo - Descending aorta; FP - False positive; LV - Left ventricle; RV - Right ventricle.

Changes in anatomy also resulted in distinct variations in the TAWSS distribution along the fetal arch anatomy, as illustrated in Fig. 6A. Transitioning towards a more CoA shape resulted in a progressive increase in TAWSS at the posterior wall of the aortic isthmus. Due to retrograde flow through the aortic isthmus, a flow split region on the posterior wall was created with a characteristic “TAWSS split pattern”, where a low TAWSS area is surrounded by a high WSS area (see red arrows in the posterior aortic isthmus wall on Fig. 6A and C.). In the arterial duct, the transition towards a more CoA shape resulted in higher TAWSS in the junction with the inner curvature of the aortic arch. Conversely, as the shape changed towards a healthier anatomy, these two patterns of TAWSS were gradually reduced.

## 4 Flow changes

In the second experiment, independently changing the inlet boundary conditions also induced nearly identical haemodynamic changes across the two representative shape phenotypes, as illustrated in Fig. 4B. Changing the ventricular output towards a more CoA configuration, with decreased LV flow and increased RV flow, resulted in the gradual increase in retrograde flow through the aortic isthmus during the entire cardiac cycle (see Table 2). In the extreme CoA flow inlet conditions (i.e., 85/15 % flow from RV and LV, respectively), retrograde flow eventually became evident. On the other hand, progressive changes towards a more balanced cardiac output resulted in increased antegrade flow without any retrograde flow during the entire cardiac cycle (see the bottom row in Fig. 4B and Table 2. Fig. 5B shows the velocity waveforms at the aortic isthmus for each of the simulated haemodynamic conditions, showing how the increased disparity between RV and LV output resulted in increased retrograde flow with an earlier peak of reverse flow, particularly in the confirmed CoA case.

**Table 2:** Comparison of the time-averaged wall shear stress (TAWSS) and flow rate *Q* at the aortic isthmus when moving along the flow axis (i.e., changing the balance between right and left ventricular output).

	Flow changes - % of flow from RV/LV			
	**≈ 55/45 %**	**≈ 65/35 %**	**≈ 75/25 %**	**≈ 85/15 %**
TAWSS	**23.5 ± 15.5**/10.5 ± 5.8	**10.9 ± 6.6**/6.1 ± 4.1	**9.2 ± 5.4**^b^/3.3 ± 3.8^a^	**27.5 ± 15.4**/4.4 ± 4.7
**TAWSS**_*max*_	**55.4**/24.0	**29.1**/17.7	**40.0**^b^/23.1^a^	**64.1**/28.0
**Q**_*ist*_	**131.8**/247.0	**69.6**/147.6	**42.7**^b^/67.5^a^	**105.7**/70.9
**Q**_*istretrograde*_	**−1.3**/0.0	**−7.0**/0.1	**−33.5**^b^/-1.7^a^	**−103.8**/-57.4

The bold values correspond to the simulations starting from the true positive baseline. TAWSS shows the mean ± standard deviation of the aortic isthmus region.

TAWSS_*max*_ is the maximum TAWSS. Q_*ist*_ is the total flow rate through the aortic isthmus. Q_*istretrograde*_ is the retrograde flow rate. TAWSS values are shown in Pa; *Q* values in mL/min.

a Shows the false positive baseline results.

b The true positive baseline ones.

Differences in TAWSS as a result of the changes in haemodynamic conditions are shown in Fig. 6B and Table 2, again showing nearly identical findings to shape changes above. In the FP shape phenotype, an increase in RV blood flow resulted in decreased TAWSS in the aortic isthmus and increased TAWSS in the arterial duct. Transitioning towards healthier splits with increased LV flow resulted in the opposite pattern. Similarly, in the confirmed CoA shape phenotype, changes towards a healthier haemodynamic configuration resulted in decreased TAWSS at the arterial duct and increased TAWSS at the aortic isthmus, with a focal point of high TAWSS on its posterior wall. Transitioning towards a more CoA configuration progressively reduced the high TAWSS spot on the posterior wall, ultimately leading to the “split TAWSS pattern” described before (see red arrows in Fig. 6C.)

## 5 Discussion

Anatomical arch remodelling towards a CoA phenotype affects flow at the aortic isthmus, with increased retrograde flow through it and the appearance of a “split TAWSS pattern” at the expected location of the posterior aortic isthmus shelf - see Fig. 4, Fig. 6. Independently changing ventricular output, i.e. simulating right ventricular dominance, leads to a nearly identical flow and TAWSS pattern at the isthmus. The appearance of these distinctive flow features when independent changes in flow conditions and anatomy are applied suggests the existence of a fundamental link between anatomy and function at this critical interface. Our work shows a first step towards the use of digital twins of the fetal circulation for improved mechanistic understanding of the onset of CoA before birth and its potential for the exploration of the longitudinal changes in the homeostatic equilibrium ruling vessel development, and thus risk of CoA, before birth.

### 5.1 The interplay between fetal arch shape and flow in CoA

Arterial remodelling results from the dynamic interplay between haemodynamic forces, structural vessel adaptation and genetic factors. Recent studies have underscored the significance of fetal arch shape in the diagnosis and pathophysiology of CoA [12,13]. Nevertheless, our understanding of the mechanisms associated with these shape changes has been constrained by the limited availability of prenatal three-dimensional flow data. Our study is based on a uniquely rich dataset where MRI data of fetal flow and anatomy were acquired simultaneously. Statistical shape analysis and flow metrics from this cohort allowed us to select two representative cases of false positive and true positive diagnoses. This allowed us to build a novel synthetic cohort spanning the flow and shape profiles of true CoA and false positive cases to perform a parametric study on the mutual effect of independent flow and shape variations. We demonstrated that instantaneous perturbations in both shape and cardiac output distribution from a baseline condition can significantly affect the flow dynamics at the junction between the arterial duct and aortic isthmus. In particular, two flow characteristics in the aortic isthmus, the retrograde flow and the “split TAWSS pattern” emerged as key features of interest.

The aortic isthmus is a very particular arterial shunt in the fetal circulation due to its location between the origin of the left subclavian artery and the aortic end of the arterial duct [29]. Its haemodynamic pattern reflects the complex interaction between cardiac output distribution and downstream/upstream vascular resistance, providing significant information about fetal haemodynamic adaptation, compromise and structural pathology [30]. Previous studies have associated the appearance of a peak of reverse flow at the end of systole in the aortic isthmus with delayed and longer ejection of the right ventricle as compared to the left [29,31,32]. In our study, both a CoA shape phenotype (e.g. with a narrower aortic arch) and right ventricular output dominance led to a shift from antegrade to retrograde flow through the aortic isthmus, from 0.0 mL/min or minimal retrograde flow to up to 103.8 mL/min in the most extreme diseased conditions (see the flow rates through the aortic isthmus in Table 1, Table 2, and the red arrows in Fig. 4, Fig. 5). Right ventricular dominance is a common characteristic of normal fetuses that increases with gestational age [33]. Therefore, its influence on the retrograde flow pattern in the aortic isthmus, and its specificity in CoA, remain an open question.

Concurrently with the shift to retrograde flow, both shape and flow changes led to an increase in TAWSS in the isthmus wall (see mean and max TAWSS results in Table 1, Table 2). Ultimately, due to the flow split at the bifurcation, this culminated in the “split TAWSS pattern” on the posterior isthmic wall, where a sharp transition in.

TAWSS magnitude occurs from a circular area of near-zero values to a surrounding region subjected to high shear, as shown in Fig. 6C. Interestingly, this consistent pattern was observed not only in the true positive CoA extreme anatomy but also in synthetically generated extreme CoA configurations from the FP baseline. This underscores the significance of this specific TAWSS pattern as a potential hallmark of both anatomical and haemodynamic changes associated with CoA.

**Fig. 7:**
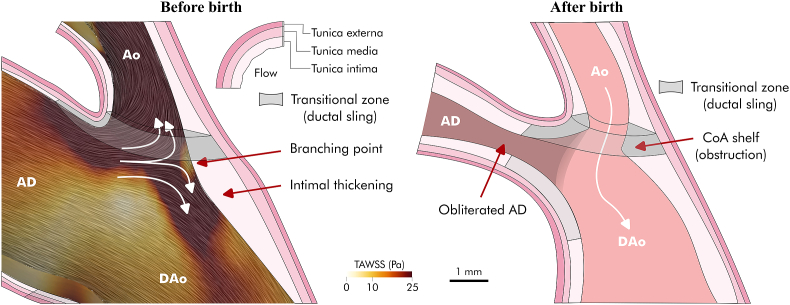
Pathophysiological mechanisms driving CoA before and after birth. Before birth, the split of ductal flow in the arterial junction results in a branching point apex with low time-averaged wall shear stress (TAWSS), which can trigger the thickening of the vessel's intimal layer and the anatomical remodelling of the arch. This remodelling can result in partial encircling of the aortic isthmus with arterial duct cells (ductal sling or transitional zone). After birth, the arterial duct closes, and the intimal thickening leads to a shelf that obstructs the arterial lumen. Ductal cells can also contribute to the obstruction. AD - Arterial duct; Ao - Ascending aorta; DAo - Descending aorta.

Our results show that, in the most severe CoA conditions, the bifurcation of arterial duct flow into the aortic isthmus and the descending aorta resulted in a branching-point apex with low WSS in the posterior wall of the aortic isthmus, i.e. the “split TAWSS” pattern illustrated in Fig. 6. This split pattern due to impinging flow has been previously related to the formation of intimal pads [34] and agrees with the findings by Hutchins [4] and Rudolph et al. [5] in support of the haemodynamic theory. Abnormal low levels of WSS can trigger endothelial dysfunction and inflammation, leading to the accumulation of lipids, inflammatory cells and smooth muscle cells within the arterial wall's intimal layer. Smooth muscle cells migrate from the tunica media to the intima, where they initiate proliferation. The proliferation of smooth muscle cells and extracellular matrix proteins like collagen contribute to intimal thickening and the eventual formation of intimal cushions [[35], [36], [37]]. These changes can further narrow the lumen and may result in the development of a posterior shelf (see Fig. 7), a hallmark of CoA observed after birth.

The main anatomical signature difference in the disease axis from FP to a true CoA is the transition of a “balanced Y-junction” between the arterial duct and the aortic isthmus, to a dominance and alignment of the arterial duct and DAo segments to which the aortic isthmus inserts to. The proximal displacement of the aortic isthmus and its high insertion angle into the arterial duct have been suggested as key anatomical features in CoA, and were found to be significantly associated with the net amount of blood flow through the aortic isthmus [12,13]. The proximal displacement of the aortic isthmus and its steep insertion on the bifurcation could be explained as a remodelling adaptation to optimise the retrograde flow through the aortic isthmus towards the left subclavian artery.

The recent evidence showing significant fetal arch shape differences before birth [12,13], and the association between CoA and altered haemodynamic conditions resulting from reduced flow through the left heart [2], have raised questions about the role of the ductal tissue extension in the onset of CoA before birth, as suggested by the Skodaic theory. Most histological studies supporting the Skodaic theory have been based on resection samples from neonatal subjects [7,[9], [10], [11],^38^,^39^]. Consequently, the observed presence of ductal cells on aortic media after birth may be a consequence of pathophysiological mechanisms initiated in utero, rather than the primary cause of CoA.

The embryonic development of the aorta, driven by the mechanisms triggered by the RV/LV flow split, could explain the presence of ductal tissue in aortic media that supports the Skodaic theory. In a histological study by Ho

and Anderson [40], the presence of a ductal sling in CoA samples was described as a diaphragmatic-like structure at the junction between the isthmus and the descending aorta. This observation led to their speculation that the ductal tissue sling may represent the original distal wall of the embryonic sixth left arch, with the arterial duct and the descending aorta forming a common channel of structural continuity and the isthmus entering it. This description agrees with the characteristics observed in our extreme CoA shape phenotype and the previously described mechanisms. Depending on the extent of the anatomical remodelling, there may exist variable amounts of original ductal tissue surrounding the remodelled aortic isthmus insertion, forming the aforementioned ductal sling or transitional zone with both ductal and aortic cells (see Fig. 7). Several histological studies [7,8,10,11,38,39] have described a high degree of variability in the presence and extent of impaired elastic fibers in the aortic media (i.e., the ductal sling), with some cases having an incomplete circular extension of ductal media at the arterial junction. This variability in extent of the ductal sling could be explained by the extent to which the arterial duct and DA form a common channel.

Our results also revealed an abnormal increase in the WSS in the junction between the arterial duct and the proximal aortic isthmus side, which could result in structural and functional endothelial cell changes, such as the migration of smooth muscle cells from the ductal media to the intimal layer, ultimately exacerbating the previously described mechanisms. Furthermore, after birth, flow disturbance at the CoA site due to the altered anatomy can lead to secondary intimal proliferation, which can further narrow the residual lumen [34,38].

### 5.2 Future work

Whilst we have shown a clear mutual relationship between flow and anatomy at the aortic-ductal junction, the underlying causative factor(s) of these changes remain unknown. Our statistical shape analysis enables encoding anatomical changes using the coefficients of the first 10 shape modes, while patient-specific CFD models provide the associated WSS distribution at the ductal insertion point. Our work represents a first step in digital twins to predict the longitudinal changes in the homeostatic equilibrium that rule vessel development in CoA before birth. Longitudinal imaging data throughout the second and third trimesters will allow us to update the patient-specific digital twin at different gestational ages, providing causal insights about whether changes in flow alter arch shape or vice versa. This could help elucidate how shape and flow might act as triggers and mutually reinforcing mechanisms in a progression cycle leading to CoA.

Given the complex dynamic nature of haemodynamic and structural adaptation during fetal life, further simulation studies with adaptive vessel growth in response to mechanical stimuli and the exploration of fluid-structure interaction (FSI) simulations could also be performed to understand how the interplay between shape and flow alters the homeostatic equilibrium ruling arterial development in utero. For instance, such models can allow the study of cyclic stretch due to pressure changes and its role in the CoA pathophysiology. Additionally, such models can go beyond a purely mechanistic investigation, offering the possibility to identify patient-specific disease trajectories. The integration of digital twin approaches with novel anatomical and functional imaging techniques [41,42] holds promise for enhancing the differentiation between true and false positive diagnoses before birth.

Furthermore, CoA has been suggested to have a genetic foundation, with candidate genes such as NOTCH1 (Neurogenic locus notch homolog protein 1), MCTP2 (multiple C2 and trans-membrane domain containing 2), and FOXC1 (forkhead box C1) identified as potential players [[43], [44], [45], [46]]. Understanding the underlying genetic mechanisms of CoA would improve our knowledge about the CoA pathophysiology. Given the potential heterogeneity in the mechanisms driving CoA, and the ethical and methodological considerations that limit the study of the fetal arch in utero, integrating our proposed approach with genetic data has the potential to further improve risk stratification in these cohorts.

Lastly, there is a need for multidisciplinary studies where computational approaches are combined with clinical, experimental and histological data to further advance our understanding of the CoA pathophysiology.

## 6 Limitations

This study has some limitations. First, our study is based on a relatively small sample size, which may not fully capture the full spectrum of anatomical and haemodynamic variations in CoA. Fetal data is scarce, and thus, the

extension of the current pipeline to study more anatomies in detail is warranted.

Second, the use of smooth patient-specific anatomical reconstructions resulting from the linear combination of the 10 first PCA modes could alter the presence of secondary structures. This limitation lies in the challenge of obtaining high-resolution patient-specific anatomical data, particularly in the small vessels of the fetus. Additionally, given that pressure measurements can not be obtained during fetal life in clinical practice, we could not validate the systolic and diastolic pressures from our models.

Third, we did not consider the combined influence of changes in flow and shape with alterations in cerebropla-cental resistance of parameters such as the time-to-peak velocity at the inlets or the ejection time, which require further investigation.

Additionally, variations in the position of brain arteries, notably the distance between the left subclavian artery and the left carotid artery, were not accounted for, despite their known associations with CoA [2].

Finally, we assumed laminar flow conditions in all our CFD simulations. However, high velocities and sudden changes in vessel diameter might result in transient or turbulent flow. Future studies could explore using more detailed turbulence models to capture better the potential turbulence generation [47].

## 7 Conclusions

A haemodynamic phenotype characterised by the appearance of (1) retrograde flow through the aortic isthmus and (2) a “TAWSS split pattern” at the posterior wall of the aortic isthmus was consistently observed by perturbing baseline conditions with either (A) anatomical changes towards CoA or (B) right ventricular dominance with increased ventricular flow output. The consistent link between form and function supports both of the major theories regarding the aetiology of coarctation in fetal life and the rationale for novel biomarkers integrating digital twin approaches with clinical data to improve risk prediction in CoA.

## Funding

This work was supported by the 10.13039/100010269Wellcome Trust IEH Award (102431) (iFIND project), core funding from the 10.13039/501100023312Wellcome/EPSRC Centre for Medical Engineering (WT203148/Z/16/Z) and the 10.13039/501100000833Rosetrees Trust (A2725). The research was funded by the National Institute for Health Research (NIHR) Biomedical Research Centre based at Guy's and St Thomas' 10.13039/100030827NHS Foundation Trust and King's College London and supported by the NIHR 10.13039/501100018835Clinical Research Facility. The views expressed are those of the author(s) and not necessarily those of the NHS, the NIHR, or the Department of Health. D.F.A. Lloyd holds a British Heart Foundation Intermediate Clinical Research Fellowship (FS/ICRF/22/26028). P. Lamata held a Wellcome Trust Senior Research Fellowship (209450/Z/17/Z).

## Data availability

Source data for this study are not publicly available due to privacy or ethical restrictions. The source data are available to verified researchers upon request by contacting the corresponding author.

## CRediT authorship contribution statement

**Uxio Hermida:** Writing – original draft, Visualization, Software, Methodology, Investigation, Formal analysis, Data curation, Conceptualization. **Milou P.M. van Poppel:** Writing – review & editing, Data curation. **Malak Sabry:** Writing – review & editing, Software, Methodology. **Hamed Keramati:** Writing – review & editing, Software, Methodology. **Johannes K. Steinweg:** Writing – review & editing, Data curation. **John M. Simpson:** Writing – review & editing, Data curation. **Trisha V. Vigneswaran:** Writing – review & editing, Data curation. **Reza Razavi:** Writing – review & editing, Supervision, Data curation. **Kuberan Pushparajah:** Writing – review & editing, Supervision, Data curation. **David F.A. Lloyd:** Writing – review & editing, Supervision, Data curation. **Pablo Lamata:** Writing – review & editing, Supervision, Methodology, Investigation, Conceptualization. **Adelaide De Vecchi:** Writing – review & editing, Supervision, Methodology, Investigation, Conceptualization.

## Declaration of competing interest

None declared.
