# Supplementary material for: The onset of coarctation of the aorta before birth: Mechanistic insights from fetal arch anatomy and haemodynamics
Source: Comput Biol Med. 2024 Nov;182:109077. doi: 10.1016/j.compbiomed.2024.109077 (PMC11846778; doi:10.1016/j.compbiomed.2024.109077)
Supplement: Multimedia component 1 [file mmc1.docx]

# Supplementary Material - The Onset of Coarctation of the Aorta Before Birth: Mechanistic Insights from Fetal Arch Anatomy and Haemodynamics

Uxio Hermida, Milou P.M. van Poppel, Malak Sabry, Hamed Keramati, Johannes K. Steinweg, John M. Simpson, Trisha V. Vigneswaran, Reza Razavi, Kuberan Pushparajah, David F.A. Lloyd, Pablo Lamata, and Adelaide De Vecchi

***3D shape reconstruction for CFD study***

In constructing the SSM, we prioritised accuracy and robustness by using centrelines to capture the 3D shape of the fetal arch, as opposed to relying on the original 3D surfaces from semi-automatic segmentations of the fetal CMR images. This choice stems from the susceptibility of the latter to significant surface irregularities. To align with the anatomical signature captured by our LDA axis, which incorporates shape changes captured only by the first 10 PCA modes, and to exclude irrelevant shape changes from the sensitivity analysis that could lead to altered haemodynamic patterns, we opted to construct the 3D anatomies for CFD using reconstructed surfaces from these 10 PCA modes. Each patient-specific centreline, denoted as *x_i_*, can be approximated by the linear combination of the first 10 PCA modes Φ as

10

*x_i_ x*¯ + ∑ *b_m_*Φ*_m_* (2)

≈

*m*=1

where *x*¯ is the average shape from the population, and *b_m_* the case-specific shape coefficients along each PCA mode Φ*_m_*. A 3D shape can then be reconstructed. This approach ensures that the shape changes in our sensitivity analysis remain aligned with our clinical question, avoiding the inclusion of spurious shape features.

The reconstructed 3D shape from the first 10 PCA modes lacks representation of the left and right pulmonary arteries, alongside the arterial segments extending to the upper body and brain. To address this, a new set of centrelines using the patient-specific anatomy was extracted, encompassing all segments essential for the CFD study. Outlets were automatically identified and extended to five times their diameter - a standard practice to enhance the numerical stability in CFD simulations. The intersection between the surface reconstructed solely with the main segments and the centreline containing all segments was used to locate and clip the centrelines corresponding to the additional segments. Merging these extra centrelines with the main centreline reconstructed with the PCA modes ultimately yielded the 3D surface for CFD simulations.

***0D lumped model of the fetal circulation***

We modelled the fetal circulatory system with a 0D model that includes 8 arterial segments and 6 outlets: left and right pulmonary arteries, brain arteries and descending aorta (see Supplementary Fig. 1). Each arterial segment is represented with a resistance *R* = 8*µl/πr*^4^ in series with an inductance *L* = *ρl/πr*^2^. The values of length *l* and radius *r* for each segment were derived from the 3D anatomy derived from the first 10 PCA modes. Literature values were used for the blood viscosity *µ* and blood density *ρ*[1](#_bookmark54). Each outlet was defined by a proximal resistance *R_p_* connected in series with a parallel combination of a distal resistance *R_d_* and a capacitor *C* (i.e., three-element Windkessel model). *R_p_* was defined to equal the characteristic impedance of the feeding artery at high frequencies *f* as

*R_p_* = *real*{*Z_c_*} = √*Z_T_ Z_L_* (3)

where *Z_T_* = 1*/ j*2*π f C* and *Z_L_* = *R* + *j*2*π f L*. *R*, *L* and *C* are the electrical components of the feeding artery. Since a rigid wall model is used for 3D CFD, the capacitance was approximated as *C* = 3*πr*^3^*l/*2*Eh*, where *h* is the wall thickness of the artery, assumed to be 10% of the radius, and *E* the Young’s Modulus from[^1^](#_bookmark54). The model was built with Simulink, MATLAB (2022a, The MathWorks Inc., Natick, MA). The values of *R_p_* and *C* for each outlet were calibrated for each of the two baseline cases to minimise the discrepancy between the 0D model output and 2D PC-MRI data. Values for each outlet were first initialised from previous research[^1^](#_bookmark54), and then optimised to find the *R_p_* and *C* values that minimise the sum of the relative errors between the model output and combined cardiac output (CCO) distribution, flow rate at the DAo, time-to-peak velocity, and theoretical systolic and diastolic blood pressures, calculated following the equations from Struijk et al.[^2^](#_bookmark55):

*P_sys_* = 1*.*06 · *GA* + 15*.*91 (4)

*P_sys_* = 0*.*64 · *GA* + 2*.*47 (5)

where GA is the gestational age of the fetus in weeks: 30.3 weeks - confirmed CoA case; 31.3 weeks FP case.

The optimised 3WK parameters for the two baseline simulated cases are shown in Table [S1](#_bookmark51).

***Mesh-independence study***

A mesh-independence study was conducted using a Grid Convergence Index (GCI) analysis to systematically estimate the discretisation error and quantify the convergence behaviour. The GCI was calculated for the mean


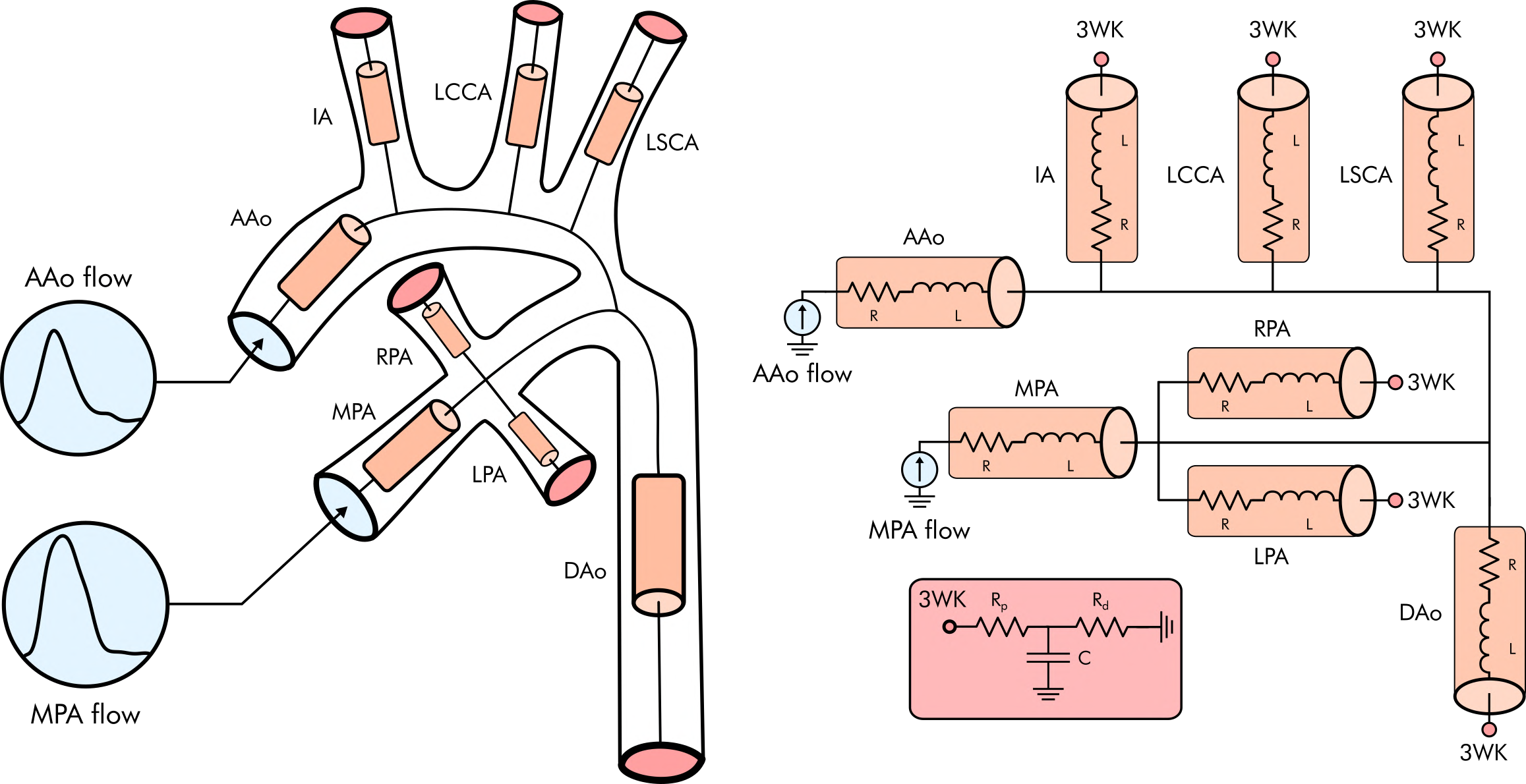


**Figure S1.** 0D lumped parameter model of the fetal circulation. The left panel shows a schematic representation of the fetal circulation. The right panel shows the equivalent 0D lumped parameter model electrical circuit where each arterial segment is modelled with a resistance (*R*) in series with an inductance (*L*). Each peripheral vascular bed is modelled with a three-element Windkessel model, consisting of a proximal resistance *R_p_*, a capacitor *C*, and a distal resistance *R_d_*. On the left panel, AAo flow represents an example flow rate waveform at the ascending aorta (AAO) from 2D phase-contrast magnetic resonance imaging data. MPA flow represents the flow waveform at the main pulmonary artery (MPA). Red blocks in the left and right panels represent the peripheral vascular beds. Orange blocks represent each arterial segment. AAO: Ascending aorta; DAo: Descending aorta; C: Capacitor; IA: Innominate artery; L: Inductance; LCCA: Left common carotid artery; LSCA: Left subclavian carotid artery; LPA: Left pulmonary artery; MPA: Main pulmonary artery; RPA: Right pulmonary artery; 3WK: Three-element Windkessel model; *R_p_*: Proximal resistance; *R_d_*: Distal resistance.

## Artery False Positive baseline True Positive baseline

*R_p_* (Pa·s/m^3^) *R_d_* (Pa·s/m^3^) *C* (m^3^/Pa) *R_p_* (Pa·s/m^3^) *R_d_* (Pa·s/m^3^) *C* (m^3^/Pa) DAo 1*.*21 × 10^8^ 4*.*45 × 10^8^ 3*.*68 × 10^−11^ 1*.*60 × 10^8^ 5*.*19 × 10^8^ 5*.*77 × 10^−11^

LCCA 7*.*95 × 10^8^ 2*.*14 × 10^9^ 1*.*81 × 10^−10^ 1*.*20 × 10^9^ 1*.*37 × 10^9^ 4*.*00 × 10^−11^

IA 8*.*73 × 10^8^ 2*.*14 × 10^9^ 1*.*81 × 10^−10^ 1*.*33 × 10^9^ 1*.*37 × 10^9^ 4*.*00 × 10^−11^

LSCA 4*.*38 × 10^8^ 4*.*54 × 10^9^ 4*.*60 × 10^−10^ 5*.*99 × 10^8^ 4*.*61 × 10^9^ 5*.*63 × 10^−10^

RPA 3*.*52 × 10^8^ 3*.*81 × 10^8^ 4*.*97 × 10^−9^ 3*.*54 × 10^8^ 1*.*21 × 10^9^ 6*.*63 × 10^−11^

LPA 4*.*88 × 10^8^ 3*.*81 × 10^8^ 4*.*97 × 10^−9^ 5*.*46 × 10^8^ 1*.*21 × 10^9^ 6*.*63 × 10^−11^

**Table S1.** Three-Element Windkessel Parameters for the two baseline simulated cases. *R_p_* represents the proximal resistance; *R_d_*, the distal resistance; *C*, the capacitor. DAo: Descending aorta; LCCA: Left common carotid artery; IA: Innominate artery; LSCA: Left subclavian carotid artery; RPA: Right pulmonary artery; LPA: Left pulmonary artery.

time-averaged wall shear stress (TAWSS) in the aortic isthmus region of the false positive baseline case. The mean TAWSS values obtained from simulations with fine, medium, and coarse meshes are summarised in Table [S2](#_bookmark52), along with the average element volumes for each mesh. The meshes were generated with a refinement ratio of 1.2.

The results indicate that the medium mesh offers a good balance between computational cost and numerical accuracy, with a GCI of 0.54%, which is significantly lower than the GCI for the coarse mesh. Figure [S2](#_bookmark53) provides a graphical representation of the TAWSS with respect to the average element volume size of the mesh.

Mesh discretisation Average Volume (*m*^3^) TAWSS (*Pa*) *ε* (%) GCI (%) Fine 1*.*4 × 10^−12^ 3.473 - -

Medium 2*.*4 × 10^−12^ 3.491 0.52 0.54

Coarse 4*.*1 × 10^−12^ 3.530 1.10 1.15

**Table S2.** Mesh-independence study for the mean time-averaged wall shear stress (TAWSS) in the aortic isthmus region of the false positive baseline case. *ε* represents the approximate relative error. GCI: grid convergence index.

3*.*55

Mean TAWSS

3*.*53

3*.*51

Wall Shear Stress

3*.*49

3*.*47

3*.*45

10−12 10−11*.*7 10−11*.*4

Average Volume Size (*m*^3^)

**Figure S2.** Mean time-averaged wall shear stress (TAWSS) in the aortic isthmus region in the false positive baseline case with different mesh densities.

***Time-independence study***

To assess the independence of the results with respect to the choice of time step, we performed three simulations with three different time steps (1 · 10^−3^ *s*, 1 · 10^−4^ *s*, 5 · 10^−5^ *s*), and we assessed the flow rate in the descending aorta. Negligible differences were observed between the three simulations (see Figure **??**). The total flow rate over the

entire cycle was 438.85, 438.82 and 438.85 mL/s respectively.
